# Supplementary material for: Loss of Protein Stability and Function Caused by P228L Variation in NADPH-Cytochrome P450 Reductase Linked to Lower Testosterone Levels
Source: Int J Mol Sci. 2022 Sep 4;23(17):10141. doi: 10.3390/ijms231710141 (PMC9456303; doi:10.3390/ijms231710141)

Supplementary materials: Full gel and western blots used in analysis.

Suppl figure S1: Purification, WT POR

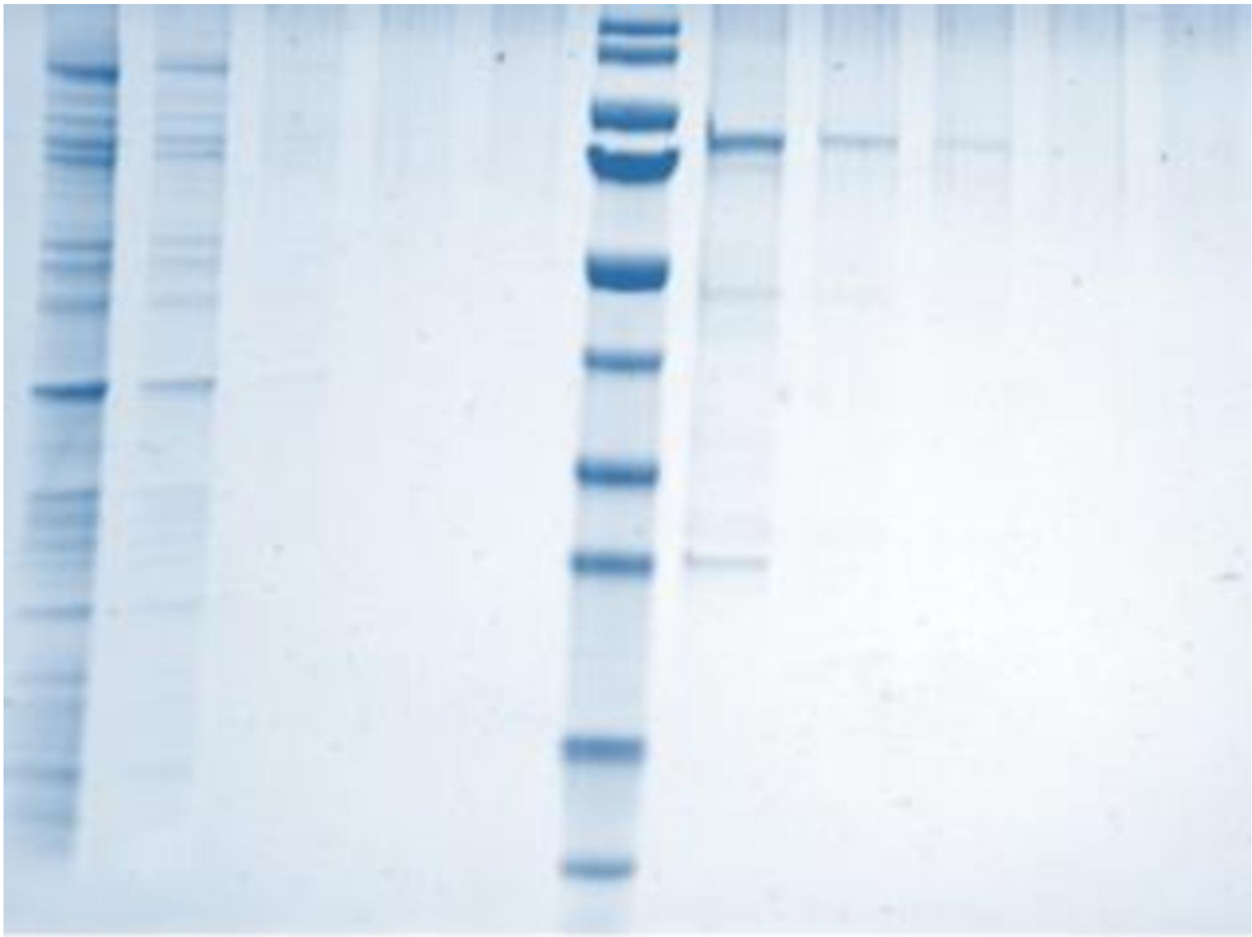

Suppl figure S2: Purification POR P228L

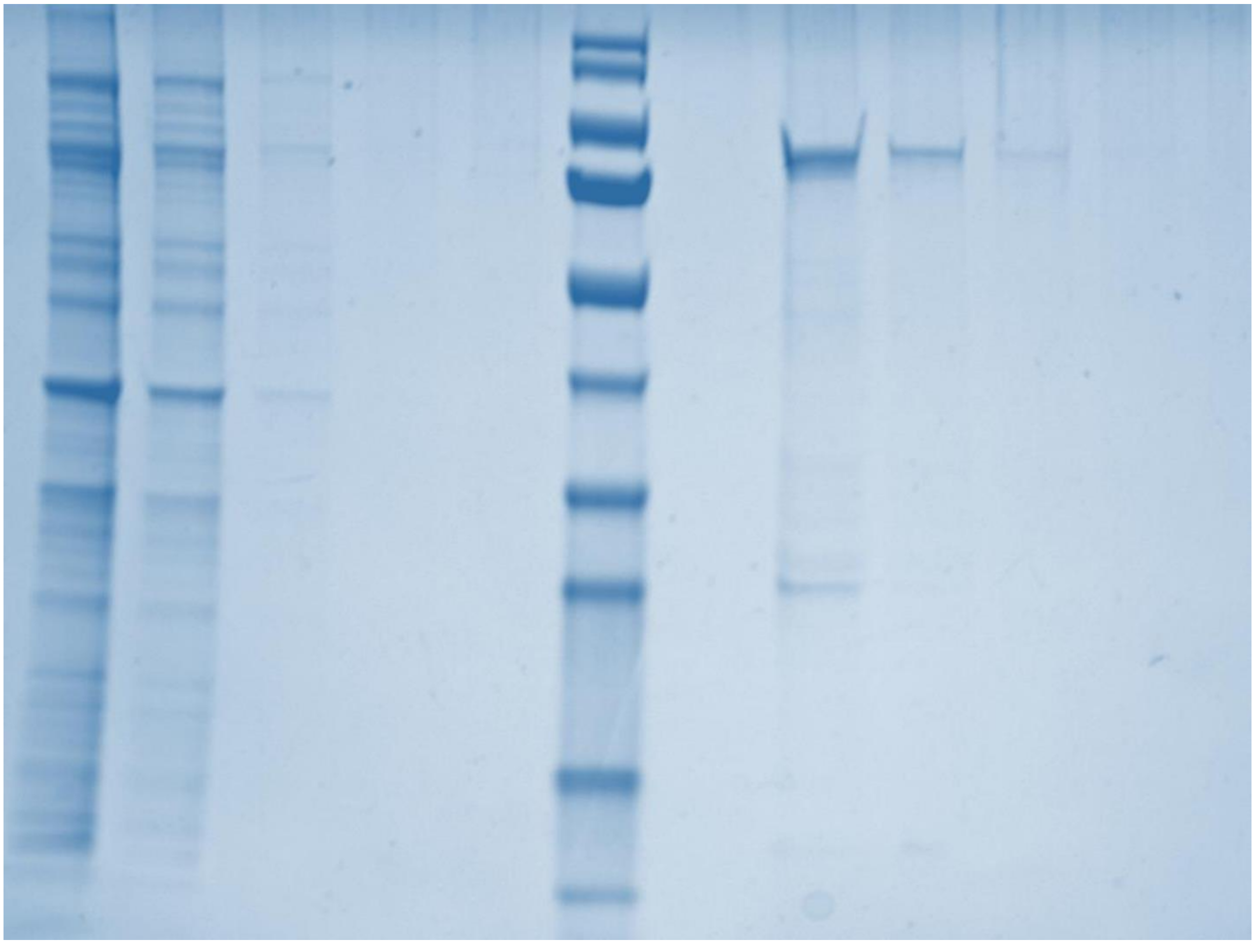

Suppl figure S3: FASTpp Western Blot: WT POR

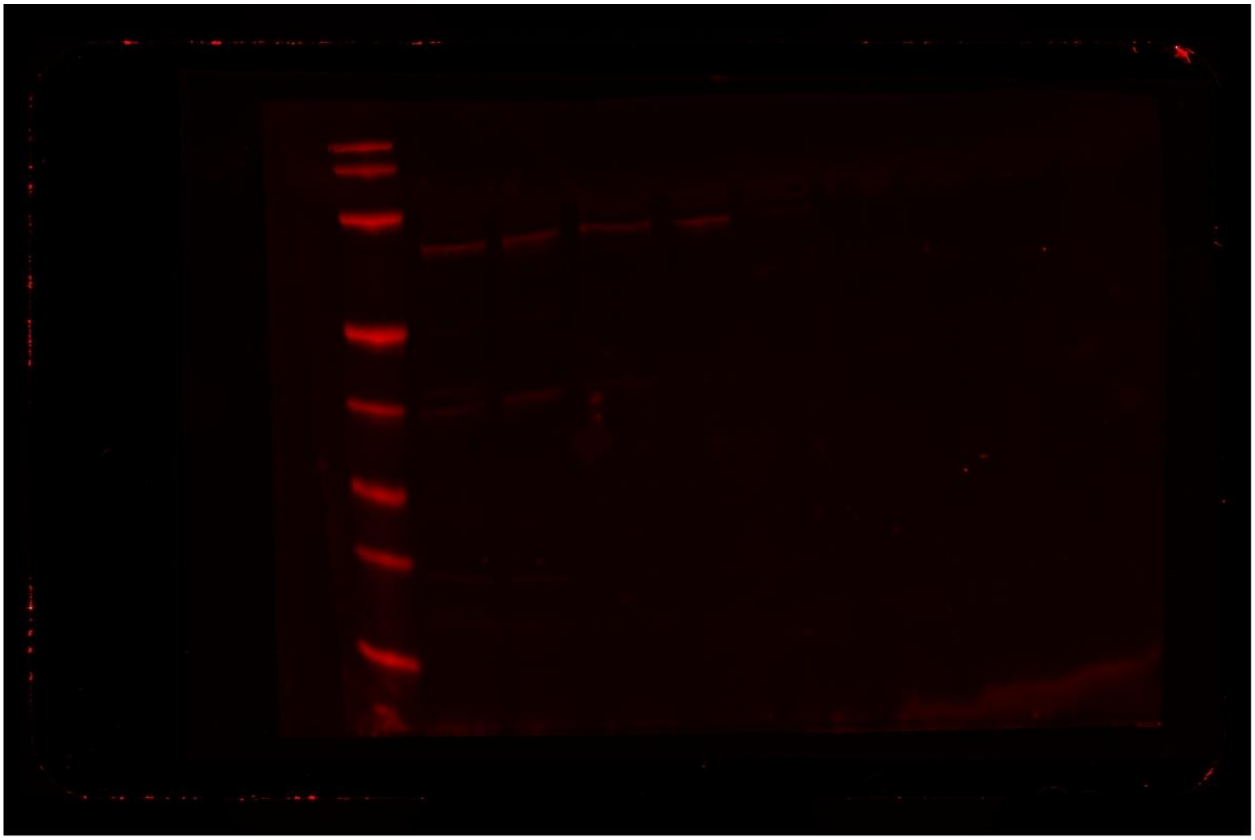

Suppl figure S4: FASTpp POR P228L Western Blot

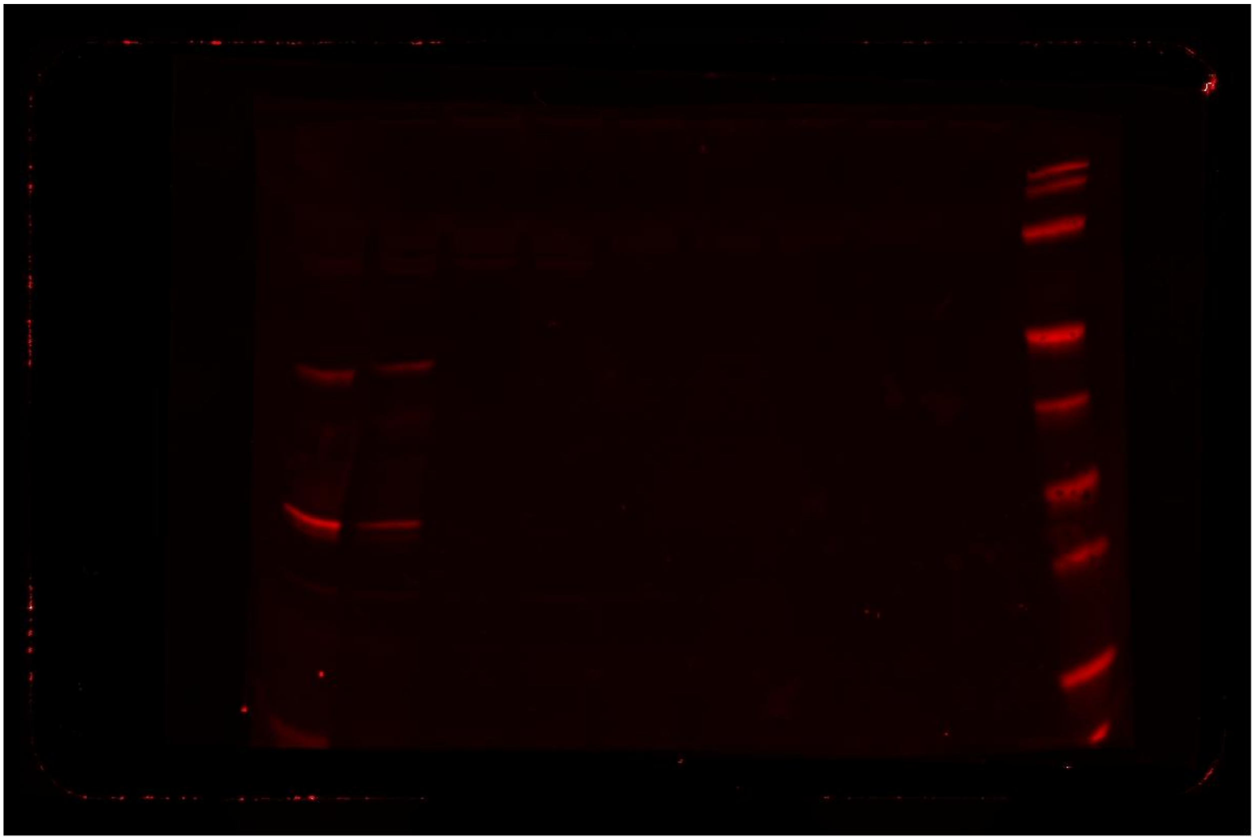

Supplement: Supplementary file 1 [file ijms-23-10141-s001.zip › ijms-1894171-supplementary.pdf]
